# Individual and Contextual Determinants of Periodontal Health in 12-Year-Old Schoolchildren in a Brazilian Capital City

**DOI:** 10.1155/2012/325475

**Published:** 2012-09-25

**Authors:** Lidia Moraes Ribeiro Jordão, Daniela Nobre Vasconcelos, Rafael da Silveira Moreira, Maria do Carmo Matias Freire

**Affiliations:** ^1^School of Dentistry, Federal University of Goias, 74605-220 Goiania, GO, Brazil; ^2^Department of Public Health, Aggeu Magalhães Institute, Oswaldo Cruz Foundation, Ministry of Health, 50670-420 Recife, PE, Brazil

## Abstract

The study aimed to describe periodontal health status and its association with individual and contextual factors among 12-year-old schoolchildren in a midwest Brazilian capital city. This cross-sectional study included data from an oral health survey carried out in 2010 in the city of Goiania, Brazil (*n* = 2, 075) and secondary data obtained from the local health authority. Data were collected through oral clinical examinations and interviews. For assessment of periodontal status two components of the community periodontal index (CPI) were used: calculus and bleeding after probing. Dependent variable was presence of any periodontal condition. Independent individual variables were the children's sex and color/race, and their mother's level of schooling. Contextual variables were related to the schools (type and existence of toothbrushing program) and its geographic location in the health districts. Rao-Scott test and multilevel Poisson analysis were performed. The prevalence of calculus and/or bleeding was 7%. Brown color, public schools, and those located in health district with intermediate socioeconomic indicators were associated to a higher prevalence of this condition. The prevalence of adverse periodontal condition was low and the inequalities in its distribution were determined by individual as well as contextual factors related to the schools and the geographic area.

## 1. Introduction

Alongside caries, periodontal diseases remain as global oral health burdens. Periodontitis constitute a major cause of tooth loss in adults worldwide, and most children and adolescents exhibit signs of gingivitis [1]. 

This is particularly the case in underprivileged subpopulations in both developing and developed countries [2, 3]. Even in high-income countries with advanced public oral health care, inequalities in periodontal health remain a major public health issue [4]. Likely reasons to account for these prevalent diseases include genetic, epigenetic, and environmental risk factors, as well as other individual and socioeconomic determinants [1].

Adolescence can be a critical period in the individual's periodontal status. Epidemiological and immunological studies suggest that irreversible tissue damage from periodontal disease begins in late adolescence and early adulthood [5]. Gingivitis prevalence, severity and extent increase with age, beginning in the deciduous dentition and reaching a peak at puberty, followed by a limited decline in adolescence [6–8].

At a population level, plaque and calculus deposition, as well as occurrence of gingivitis and periodontitis, are slightly higher in boys than in girls [6]. Dentofacial anomalies, toothbrushing frequency, socioeconomic and psychological conditions experienced in early life and life course have also been associated with gingival bleeding in adolescents [9–11]. 

Calculus and gingival bleeding are clinical indicators of poor oral hygiene and periodontal condition [12] and have been frequently used in epidemiological studies as part of the community periodontal index (CPI) [13] or separately analyzed.

Both gingival bleeding and calculus in Brazilian adolescents have been significantly associated with race, family income, levels of schooling, and type of school attended [10, 12, 14, 15]. 

Current studies show that, regardless the socioeconomic indicator used, people who are socioeconomically disadvantaged consistently have poorer periodontal outcomes [16]. However, investigation of the influence of socioeconomic status on the etiological pathway of periodontal condition is still required to better understand its determinants. 

Moreover, global reports have emphasized that continuing surveillance of levels and patterns of risk factors is of fundamental importance to planning and evaluating community preventive activities and oral health promotion in all parts of the world [17].

In that context, multilevel analysis technique considers both people and areas on the distribution and determinants of population health, being an important approach to understand the significance of specific contexts for different individual health outcomes [18]. So far, there are very few studies investigating the influence of contextual variables using multilevel analysis in the periodontal status of schoolchildren [12, 19]. 

The present study aimed to describe periodontal health status and its association with individual and contextual factors among 12-year-old schoolchildren in a midwest Brazilian capital city.

## 2. Material and Methods

### 2.1. Type of Study and Source of Data

The present cross-sectional study was carried out in the city of Goiania, capital city of Goias State, located in the Midwest region of Brazil. The analysis included primary data from an epidemiological survey of oral health carried out in 2010. Secondary data were obtained from the local health authority in the same year. 

The oral health survey of 12-year-old schoolchildren in the urban area followed the methodology of the 2010 Brazilian National Survey of Oral Health [20]. Clinical examinations were according to diagnostic criteria established by the World Health Organization (WHO) [21]. Although data on other oral conditions were collected, only data on periodontal condition was included in the present study.

### 2.2. Ethical Aspects

The research protocol was approved by the Ethics Committee of the Federal University of Goias, Brazil (Report 226/2010) and only the schools and the children whose parents signed an informed consent participated in the study.

### 2.3. Sample

The age 12 years was chosen following the WHO recommendations for the monitoring of oral health status among children. It is generally the age at which children leave primary school, and therefore it is the last age at which a reliable sample may be easily obtained through the school system [21]. 

Sample size was calculated to be representative of the 12-year-old schoolchildren in Goiania. We used the cluster sampling technique and the sample was randomly selected in two stages. Initially, we draw the number of first stage unities (schools), followed by second stage unities (schoolchildren). 

According to data obtained from the State and the City's Education Department, the total number of 12-year-old schoolchildren enrolled in 2009 was 17,911 in 281 public and private schools. Sample size was calculated using an equation for proportions for infinite populations based on caries prevalence, using the Epi Info software, version 3.5.1. The minimum number of schoolchildren to take part in the research was 2,171, considering a confidence interval of 95%, sampling error of 2%, and caries prevalence of 65.3%. For effect of study sample design, a simplified and conservative correction was needed, multiplying the obtained sample size by 1.2 (an extra 20%). The final sample size was 2,605 schoolchildren.

To calculate the amount of schools, we used a formula that consisted of multiplication of the number of schools by the number of schoolchildren of the sample, divided by the total number of 12-year-old schoolchildren in Goiania. We then obtained a sample of 41 schools.

The sample was equitably distributed in the seven health districts in the city: Central, Eastern, Northwestern, Northern, Western, Southwestern, and Southern. Each health district comprises a geographic area with specific population, well-defined health issues, and unique interaction with health care teams.

As the total sample was of 2,605 schoolchildren, and the number obtained from the list was approximately of 2,962 schoolchildren, we opted for including all students attending the selected schools. 

### 2.4. Data Collection

Data were collected through oral clinical examinations and interviews with the children by six teams composed by one dentist and one recorder, who worked in the public health service. They were previously trained and calibrated, according to the criteria used in the 2010 Brazilian National Survey of Oral Health [20]. Inter-examiner Kappa coefficient for periodontal condition varied from 0.68 to 1.00, showing a good to excellent reproducibility. 

Intraoral examinations were carried out at the schools using a mouth mirror and a WHO periodontal probe under natural light, with the children seated in school chairs. 

For the assessment of periodontal status, two components of the Community Periodontal Index (CPI) [20] were used: calculus and bleeding. Periodontal pockets were not registered. Each sextant of the mouth was examined for calculus detected during probing and bleeding observed after probing. Index teeth were 16, 11, 26, 36, 31, and 46. In each tooth, six sites were examined, and the worst condition was recorded. Gingival bleeding was recorded from 10 to 30 seconds after probing. Recommended probe placement was of approximately 60 degrees inclination in relation to the longitudinal axis of the tooth. 

Information on demographic and socioeconomic characteristics of the participating children was also collected: sex, self-rated skin color or race, and mother's level of schooling. Self-rated skin color or race followed criteria proposed by the Brazilian Institute of Geography and Statistics (IBGE): white, black, yellow (Asian origin individuals), brown, or indigenous. Mother's level of schooling was based on completed years of study and was obtained from the children's school records.

Secondary data were: type of school (public and private), existence of toothbrushing program at the schools, and the city's health districts where the schools were located. 

The School Toothbrushing Program was created in 1992 through a partnership between the local Health and Education Secretaries. Its aim is to improve the oral health status of schoolchildren enrolled in public schools of Goiania via implementing daily toothbrushing with fluoride toothpaste after the school meal.

### 2.5. Data Analysis

The dependent variable was the prevalence of periodontal condition, featured by the presence of calculus and/or bleeding (yes or no).

Independent variables were divided into two levels of data organization: individual (schoolchildren) and contextual (schools and health districts). In the individual level, we analyzed children's demographic characteristics (sex and skin color or race), and one socioeconomic indicator (their mothers' level of schooling). Self-rated color/race was categorized in: white, black, and brown. Due to the small number of individuals who rated themselves as yellow or indigenous, these categories were excluded from the analysis. Level of mother's schooling was grouped as follows: less than eight, from eight to eleven and more than eleven, years of study. 

Contextual variables were type of school (public and private), existence of toothbrushing program at the school, and city's health district where schools were located. The seven health districts were grouped according to their socioeconomic characteristics, as informed by the local health authorities. The Health District located more centrally (Central-Campinas) presented better-off socioeconomic and health indicators than the others, which were located in the outskirts. Therefore, we have classified them in three categories: (Group I) with the best indicators (Central-Campinas); (Group II) with intermediate indicators (North, South and East); (Group III) with the worst indicators (Southwest, West, and Northwest).

Individual and contextual variables were initially described according to the prevalence of periodontal status. Rao-Scott test [22], an equivalent to chi-square test for complex samples, was used to test dependence between variables. This analysis was performed using the Stata 12 software, considering the complex sample plan and sample weights. 

**Figure 1:**
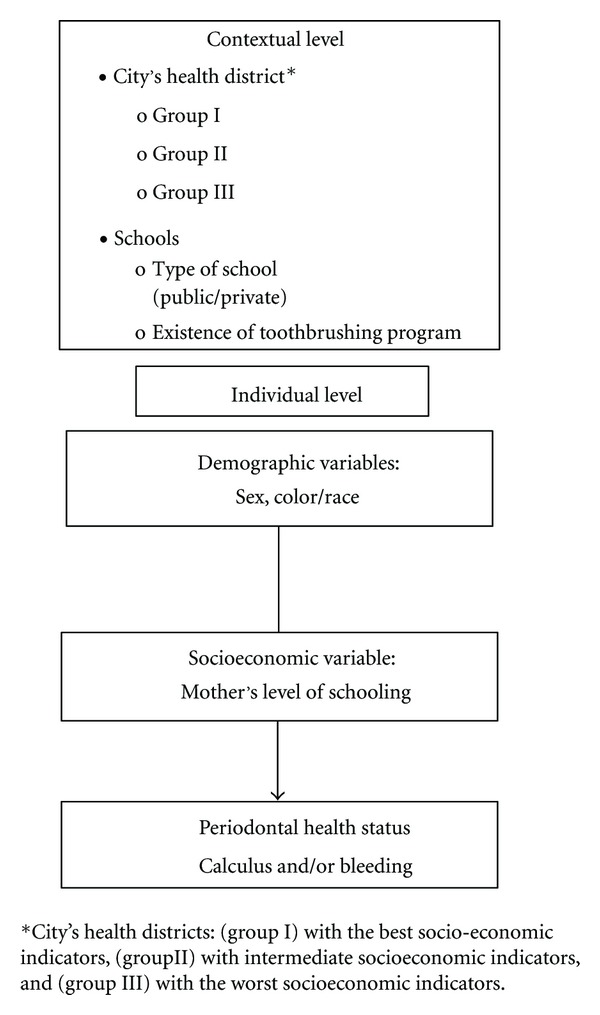
Theoretical hierarchical proposed model of the association between individual and contextual variables and periodontal health status of 12-year-old schoolchildren. Goiânia-GO, Brazil, 2010.

After that, we performed a multilevel analysis with random intercept [23], considering three levels: schoolchildren, schools, and health districts (Figure 1). Poisson log-linear regression models were adopted, with robust variance estimator, to estimate prevalence ratio as effect measure, and its confidence interval of 95% measured by Wald test. We carried out only unadjusted analysis due to collinearity between the independent variables that could jeopardize data in the multiple analysis.

In addition to considering the sample design through variance partition in each level, multilevel analysis allows for the inclusion of contextual variables of higher levels than the individual. This allows for the simultaneous approach of contextual and individual factors in the analysis. Multilevel analysis was performed using the MLwiN 2.02 software. For all analysis, we considered the 5% significance level and sample weights derived from sample design (school weights).

## 3. Results

Of the 41 schools invited to participate, 39 accepted (24 public and 15 private). Of the 2,962 schoolchildren invited to take part in the survey, 2,075 agreed and were examined (response rate = 70.0%). 

Sample was composed mainly of males (*n* = 1,053, 50.9%), those who classified themselves as brown (*n* = 1,089, 54.5%), and whose mothers had studied from eight to eleven years (*n* = 1,080, 51.2%). The majority of the students were from public schools (*n* = 1,471, 71.2%).

Table 1 presents the frequency distribution of the studied variables and the results of bivariate associations between the dependent variable and the independent variables.

Adverse periodontal conditions, such as presence of calculus and/or bleeding, were present in 140 schoolchildren, a prevalence of 7% (95%  CI = 5.3–9.2). A total of 80 individuals (4.2%, 95%  CI = 2.5–6.9) had bleeding and 85 had calculus (4.1%, 95%  CI = 2.9–5.7).

In regard to individual variables, only color/race showed statistically significant association with periodontal condition. Brown individuals had a higher prevalence of dental calculus and/or bleeding compared with the others (*P* = 0.005).

Among the contextual variables analyzed, type of school showed significant association with periodontal condition. Public schools, compared to private ones, had a higher prevalence of dental calculus and/or bleeding (*P* < 0.05).

Table 2 shows the results of unadjusted multilevel analysis. Children from schools located in health districts of group II presented a 36.9% higher probability of having adverse periodontal conditions compared to those of group I (*P* < 0.001). Those from public schools showed a prevalence of calculus and bleeding 1.54 (95%  CI = 1.14–2.08) times higher than the children from private schools (*P* = 0.004). None of the other contextual factors was significantly associated with the dependent variable.

At the individual level, schoolchildren who classified themselves as brown showed a prevalence of adverse periodontal condition 1.68 times higher than those of white color (*P* < 0.05). Sex and mother's level of schooling did not present association with the dependent variable.

## 4. Discussion

The occurrence of adverse periodontal conditions (bleeding and/or calculus) in our study was low (7.0%) compared to other Brazilian studies [7, 12] and lower than that found in schoolchildren of the same age in the city of Goiania in 2003 [15]. This may indicate an improvement on periodontal health for this age-group. The low levels of periodontal conditions generally found in Brazilian schoolchildren may be a result of the population's high awareness regarding personal hygiene, which is part of the national culture, and it is frequently associated with health and well being. The 2009 National School-Based Health Survey found that 95.2% of the adolescents reported toothbrushing frequency of twice a day or more, being higher for females [24]. 

However, our findings show that periodontal condition is associated with individual and contextual variables such as color/race, type of school and its location in the city, and confirm the persistent inequalities in the population's oral health. Higher prevalence of calculus and/or bleeding was found in those from lower socioeconomic groups. 

This study is one of the first to apply multilevel analysis to a set of data representative of adolescents attending schools in Brazil. This kind of analysis allows researchers to deal with the micro-level of individuals and the macro-level of groups simultaneously. However, issues of defining relevant contexts, specifying the relevant group-level variables, and collecting the necessary data remain a challenge [25] and that was also true in our case.

Thus, one limitation of the study was the small number of individual and contextual variables analyzed due to time constraints in the data collection period. Nevertheless, the individual and contextual factors studied here can be useful for identifying vulnerable groups and consequently contribute to a more effective and focused planning for oral health interventions.

The results did not show a significant worse periodontal status for males than females, which was different from some studies [12, 26] but in accordance with another Brazilian study [27]. The difference in findings on sex might be due to a greater concern on males' general health and body image in the whole of society over the last decade [28]. This trend could be influencing male adolescents' attitudes towards themselves.

The higher prevalence of adverse periodontal condition in brown individuals was different from previous studies that have found higher prevalence in black individuals in the state of Sao Paulo [12]. It is important to highlight that there are nearly twice more brown individuals in the population of Goias than in Sao Paulo [29], and that both groups account for lower family income, less years of study and higher illiteracy nationwide than white individuals. Therefore, both black and brown individuals are considered vulnerable groups in Brazil.

The protection of families and children's development are crucial points of attention in the public policies. In Brazil, a higher proportion of families led by black or brown individuals are couples with children younger than 14 years of age [29], stressing the importance of promoting economic and educational equality as a mean to improve families' health status as a whole.

Public schools, compared to private ones, showed higher prevalence of adverse periodontal condition. This finding has also been reported by other researchers in Brazil [12, 15, 30, 31]. Similarly, studying in rural government schools in Nepal was reported as an indicator of unhealthy periodontal status for adolescents [32]. The type of school used as an alternative indicator for socioeconomic status is seen as a feasible predictor for caries experience in epidemiological dental caries studies involving schoolchildren in the Brazilian context [31]. 

Children from schools located in health districts of group II (intermediate socioeconomic indicators) presented a higher probability of having periodontal condition compared to group I (best indicators) (*P* < 0.000). That could be partly explained by the fact that schools located in health districts with the worst oral health indicators have greater number of students covered by health and oral health public programs, which should provide prevention, education, and oral treatment. This result also shows that schools located in health districts of intermediate socioeconomic indicators should also be seen as a priority by local health authorities.

No significant difference of periodontal condition was found between schools with the School Toothbrushing Program and those without them. Systematic reviews have shown that health education interventions may result in reductions in plaque and gingival bleeding in the short-term, but it is yet unknown if these beneficial changes are sustained in the long run [33]. It is therefore recommended that oral health programs should focus on raising awareness about the issues that affect oral health and on promoting empowerment [34] so that individuals develop autonomy to make healthier choices and adopt healthier lifestyles. The present study did not aim to evaluate the effectiveness of the toothbrushing program carried out in the schools, so other studies using appropriate methodologies are needed to investigate its impact on the population. 

## 5. Conclusions 

The prevalence of adverse periodontal conditions in 12-year-old schoolchildren was low and was associated with individual and contextual variables. The inequalities in its distribution were mainly determined by the adolescents' color/race, type of school, and its location in the city. Appropriate strategies addressed to the areas of socioeconomic deprivation and the monitoring of school population oral health status are needed to reduce the disparities. 

## Figures and Tables

**Table 1:** Frequency distribution of periodontal conditions (calculus and/or bleeding), according to independent variables. 12-year-old schoolchildren, Goiania, Brazil, 2010.

	Presence of calculus and/or bleeding		Total *n* (%)	*P*
	Yes % (CI 95%)	No % (CI 95%)		
Individual variables				
Sex				0.121
Female	6.2 (4.3–8.9)	93.8 (91.0–95.7)	1,022 (49.1)	
Male	7.7 (6.0–10.1)	92.3 (89.9–94.0)	1,053 (50.9)	
Color/race				0.005*
White	4.8 (3.2–7.0)	95.2 (92.9–96.8)	787 (36.4)	
Black	4.9 (2.1–10.9)	95.1 (89.0–97.9)	192 (8.8)	
Brown	8.9 (6.9–11.3)	91.1 (88.6–93.1)	1,089 (54.5)	
Mother's level of schooling (years)				0.117
More than 11	5.6 (3.5–9.0)	94.4 (90.1–96.5)	427 (21.3)	
8 to 11	6.3 (5.1–7.9)	93.7 (92.1–94.9)	1,080 (51.2)	
Less than 8	9.3 (5.7–14.8)	90.7 (85.2–94.2)	568 (27.5)	
Contextual variables				
Health district (group)				0.337
I	4.7 (2.0–10.5)	95.3 (89.5–97.9)	425 (18.4)	
II	6.6 (4.5–9.5)	93.4 (90.5–95.5)	745 (37.9)	
III	8.3 (5.7–12.0)	91.7 (87.9–94.3)	905 (43.7)	
School toothbrushing program				0.640
Yes	6.4 (4.0–10.1)	93.6 (89.8–96.0)	632 (30.5)	
No	7.3 (5.2–10.2)	92.7 (89.8–94.8)	1,443 (69.50%)	
Type of school				0.039*
Private	4.6 (2.9–7.2)	95.4 (92.8–97.1)	604 (28.8)	
Public	8.0 (5.9–10.7)	92.0 (89.3–94.0)	1471 (71.2)	

Total	7.0 (5.3–9.2)	93.0 (90.7–94.6)		

*Rao-Scott test. *P* < 0.05.

95% CI: Confidence Interval at 95%.

**Table 2:** Results of nonadjusted multilevel analysis model of poisson log-linear regression for prevalence of periodontal condition (calculus and/or bleeding). Goiania, Brazil, 2010.

	Regression coefficient	Standard error	Prevalence ratio	Confidence interval 95%		*P*
Contextual variables						
District I	—	—	1.000	—	—	—
District II	0.3140	0.0840	1.369	1.161	1.614	0.000*
District III	0.4180	0.2850	1.519	0.869	2.655	0.143
Private school	—	—	1.000	—	—	—
Public school	0.4320	0.1540	1.540	1.139	2.083	0.004*
School toothbrushing program						
Yes	—	—	1.000	—	—	—
No	0.1260	0.1780	1.134	0.800	1.608	0.477
Individual variables						
Female	—	—	1.000	—	—	—
Male	0.2660	0.1490	1.305	0.974	1.747	0.074
White	—	—	1.000	—	—	—
Black	−0.0260	0.3550	0.974	0.486	1.954	0.943
Brown	0.5180	0.2560	1.679	1.016	2.773	0.043*
Mother's schooling (years)						
More than 11	—	—	1.000	—	—	—
8 to 11	0.1370	0.2340	1.147	0.725	1.814	0.558
Less than 8	0.3900	0.3770	1.477	0.705	3.092	0.301

*P < 0.05.
